# Entropy‐Mediated Crystallization Manipulation in Glass

**DOI:** 10.1002/advs.202411861

**Published:** 2024-12-16

**Authors:** Xu Feng, Guanfeng Gao, Quanhua Lin, Yongkang Yang, Jiajia Tan, Ziang Liu, Jianrong Qiu, Xiaofang Jiang, Shifeng Zhou

**Affiliations:** ^1^ State Key Laboratory of Luminescent Materials and Devices and Guangdong Provincial Key Laboratory of Fiber Laser Materials and Applied Techniques School of Materials Science and Engineering South China University of Technology Guangzhou 510640 China; ^2^ Key Laboratory of Atomic and Subatomic Structure and Quantum Control (Ministry of Education) Guangdong Basic Research Center of Excellence for Structure and Fundamental Interactions of Matter School of Physics South China Normal University Guangzhou 510006 China; ^3^ College of Optical Science and Engineering State Key Laboratory of Modern Optical Instrumentation Zhejiang University Hangzhou 310027 China; ^4^ Peng Cheng Laboratory Shenzhen 518000 China

**Keywords:** entropy engineering, nanocrystal‐in‐glass composite, nonlinear response, non‐metallic glass, structure evolution

## Abstract

The entropy mediated temperature‐structure evolution has attracted significant interest, which is used for the development of functional alloys and ceramics. But such strategy has not yet been demonstrated for development of non‐metallic glasses. Herein, the successful application of the entropy engineering concept to non‐metallic glass to manipulate its in situ crystallization process is demonstrated. The comparison of the entropy concept in alloys, ceramics, and non‐metallic glass is discussed. As a typical example, the activation and preservation of the entropy stabilized effect of a typical niobosilicate glass system at different temperatures are studied. The relation between the micro‐configurations and the entropic property is analyzed. Via the entropy engineering strategy, the crystallization of the niobosilicate glass can be manipulated. As a result, the LiNbO_3_ nanocrystal‐in‐glass (NiG) composite with high crystallinity is developed, which exhibits 8 times higher nonlinearity compared with the *β*‐BBO crystal. The developed NiG composite is demonstrated for practical application in precise measurement of the duration and phase of ultra‐short femtosecond pulse.

## Graphical abstract

The extension of the entropy engineering concept from alloys and ceramics to non‐metallic glass to manipulate its in situ crystallization process is demonstrated. Via the entropy engineering strategy, the LiNbO_3_ nanocrystal‐in‐glass (NiG) composite with high crystallinity and nonlinearity can be developed. This unique NiG composite enables to monitor the duration and phase of the ultra‐short optical pulse.

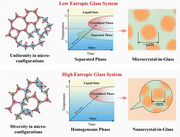

## 1 Introduction

Temperature‐structure evolution is essential to develop functional materials with specific microstructure and thus the desired mechanical, electrical, and optical properties. Recently, the strategy of entropy engineering for manipulation of temperature‐structure evolution has attracted significant interests.^[^ ^1^, ^2^, ^3^, ^4^, ^5^ ^]^ In principle, once activating entropy stabilized effect (e.g., by multi‐components mixing), the entropy of the system would predominate the thermodynamic landscape, driving a reversible transformation between a multiphase and single‐phase structure.^[^ ^6^, ^7^ ^]^ Guided by this strategy, a new class of functional materials, so called entropy stabilized materials have been developed, including alloys (crystalline and amorphous alloys)^[^ ^8^, ^9^, ^10^, ^11^, ^12^ ^]^ and ceramics.^[^ ^13^, ^14^, ^15^, ^16^ ^]^ The developed entropy stabilized materials are expected to be widely applicable for capacitive energy storage,^[^ ^17^, ^18^, ^19^, ^20^, ^21^ ^]^ fuel cell,^[^ ^22^, ^23^, ^24^, ^25^, ^26^ ^]^ and catalysis.^[^ ^27^, ^28^, ^29^, ^30^, ^31^ ^]^

Besides the crystalline materials, the entropy engineering strategy has also been guided the design of metallic glasses and even non‐metallic glasses.^[^ ^32^, ^33^, ^34^, ^35^ ^]^ A series of novel non‐metallic glasses with unique physical properties, such as ultrahigh hardness, Young's modulus, and fracture toughness has been developed. Despite the substantiate progress, the influence of entropy stabilized effects on the microstructure evolution during the whole heat history in fabricating the non‐metallic glasses still remains unexplored.^[^ ^36^ ^]^ Thus, it is compelling to consider whether the entropy stabilized effect still could be expanded to the development of functional non‐metallic glass, which fills up the missing part of the generality of the entropy engineering strategy? This is a significant and interesting question.

Inspired by research activities in the entropy stabilized inorganic materials communities, we tried to expand the concept of entropy engineering to non‐metallic glass to manipulate the microstructure evolution process. Herein, theoretically, we proposed the concept of entropy engineering strategy for non‐metallic glass, analyzing the influence of entropy on the thermodynamic landscape. Experimentally, the temperature‐structure evolution of niobosilicate based glasses with different entropic properties are discussed, guiding the fabrication of unique LiNbO_3_ nanocrystal‐in‐glass composite with high crystallinity and nanoscale grains. In addition, the giant enhanced nonlinear optical response of the developed nanocrystal‐in‐glass (NiG) composite has been evaluated, and its application in ultra‐short pulse duration and phase position measurement is also demonstrated.

## 2 Results and Discussion

### 2.1 Activation and Preservation of Entropy Stabilized Effect in Non‐Metallic Glass

For the fabrication of entropy stabilized materials, the most key factors lies on the activation of entropy stabilized effect at high temperature and preservation of the effect at low temperature.

The activation of entropy stabilized effect is a thermodynamics driven process. From the viewpoint of thermodynamics, the transformation between multiphase and single‐phase structure would be dominated by the following equation:^[^ ^6^, ^36^ ^]^

(1) $$\Delta G_{\mathrm{mix}}=\Delta H_{\mathrm{mix}}-T\Delta S_{\mathrm{mix}}$$

where ∆*G*_mix_ is the Gibbs free energy difference between the multiphase and single‐phase structure of the material system, ∆*H*_mix_ and ∆*S*_mix_ are the mixed enthalpy and entropy of the system, respectively, and *T* is the temperature of the system.

The activation of entropy stabilized effect indicates that entropy term *T*∆*S*_mix_ predominates the thermodynamic landscape, leading to the formation of stable single‐phase structure instead of multiphase structure:

(2) $$\Delta H_{\mathrm{mix}}\ll T\Delta S_{\mathrm{mix}}$$

According to Equation (2), two factors contribute equally to the activation of the entropy stabilized effect: temperature and mixed entropy of the system. The first one is the temperature. High enough temperature *T* leads to the activation of entropy stabilized effect. One can suppose the extreme example that at the temperature higher than gasification temperature, all the components would be mixed homogeneously as a single phase structure. However, restricted by the actual situation for materials fabrication, the temperature of materials fabrication would be limited. So to activate the entropy stabilized effect of a material system under the limited temperature, the second factor: the mixed entropy ∆*S*_mix_ of the system should be considered. Entropy stabilized effect is supposed to be activated under lower temperature for the materials exhibiting higher ∆*S*_mix._

While the preservation of entropy stabilized effect is a kinetic driven process. Generally, the high‐temperature fabricated materials need to be cooled down to low temperature, e.g. room temperature, eventually. In such low temperature, it is difficult to keep the thermodynamics entropy stabilized condition shown in Equation (2), leading to the transition from the single‐phase structure to multi‐phase structure during cooling process in the viewpoint of thermodynamics. The analysis indicates that under different cooling process, the micro‐structures of the entropy stabilized would be different. Thus, to preserve the entropy stabilized effect in higher degree, kinetic process must be considered. Material systems with slower relaxation properties and faster quenching process is beneficial for the preservation of the entropy stabilized effect.

Based on the above analysis, the activation and preservation of entropy stabilized effect in inorganic materials are discussed as follows. The relaxation properties and fabrication condition for typical inorganic materials are listed in **Table** **1**. Inorganic metal materials (crystalline alloys and amorphous metallic glasses) exhibit fast relaxation property due to the metal character. Inorganic crystalline ceramics are fabricated at the relative low temperature (lower than the melting point) and quenching rate. Thus, high enough ∆*S*_mix_ is supposed to overcome their drawbacks for activation and preservation the entropy stabilized effect.

**Table 1:** The relaxation properties and fabrication condition for typical inorganic materials.

Type	fabrication temperature	quenching speed	relaxation speed
Alloys	high (>melting point)	fast	fast
Ceramics	low (<melting point)	slow	slow
Non‐metallic glasses	high (>melting point)	fast	slow

According to Boltzmann law, ∆*S*_mix_ corresponds to the increase of the uncertainty of the micro‐configurations from the mixed single‐phase structure compared to the speared multiphase structure. As modeled with the ideal solid solution lattice (e.g., crystalline alloys and ceramics) or random atoms packing (e.g., amorphous metallic glasses), the ∆*S*_mix_ of these three kinds of inorganic materials could be estimated quantitatively. It is found that in such model, the ∆*S*_mix_ is originated from the uncertainty of the local element. And there are two ways to enhance the uncertainty of the local element and thus higher ∆*S*_mix_ for these three kinds of inorganic materials:

1. Adding components by partly substituting the original components equally leads to higher ∆*S*_mix_.
2. Restricted the variety of components, equal mole among all the components leads to higher ∆*S*_mix_.

Statistically, most of the metals and ceramics material systems with five equimolar or near‐equimolar components exhibit high enough ∆*S*_mix_ to activate and preserve the entropy stabilized effect under the ordinary heating and quenching process, leading to the formation of single‐phase structure. This is the core of entropy engineering strategy for temperature‐structure evolution behavior manipulation of crystalline alloys and ceramics and thus such materials composited with five equimolar or near‐equimolar components are generally defined as entropy stabilized inorganic materials.

As for non‐metallic glasses, it is considered that the entropy stabilized effect can be activated and preserved more effectively compared with alloys and ceramics. On one hand, non‐metallic glasses exhibit slow relaxation property due to their covalent or ionic character and they are fabricated via high‐temperature heating (higher than the melting point) and fast quenching process. Such situations relax the demand of ultra‐high ∆*S*_mix_ to activate and preserve the entropy stabilized effect.

**Figure 1:**
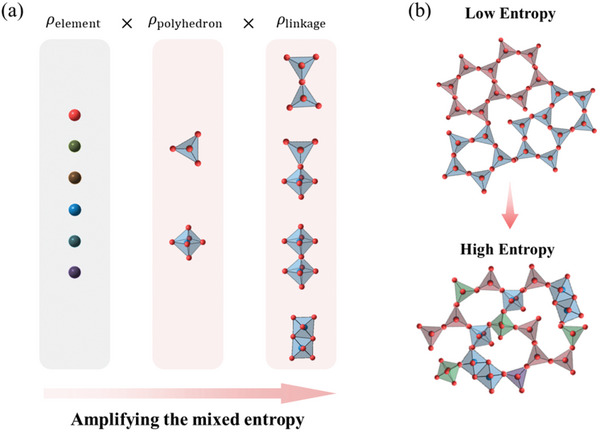
The concept of entropy engineering in non‐metallic glass. a) Amplification of mixed entropy in non‐metallic glass via component diversification. b) Activating entropy stabilized effect in non‐metallic glass.

On the other hand, sharper increase of ∆*S*_mix_ is supposed with the diversity of the components. Without an ideal solid solution lattice, the ∆*S*_mix_ cannot yet be estimated quantitatively. While without the limit of the solid solution lattice frame, the ∆*S*_mix_ is supposed to not only be originated from the uncertainty of local elements but the uncertainty of the types of the polyhedron and their linkage, leading to the amplification of ∆*S*_mix_ (**Figure** **1**a). Less component of the non‐metallic glass system is enough to activate and preserve the entropy stabilized effect, indicating that most of the ordinary non‐metallic glass might be in entropy stabilized state naturally (Figure 1b).

### 2.2 Entropy Engineering for Manipulation of Glass Formation

**Figure 2:**
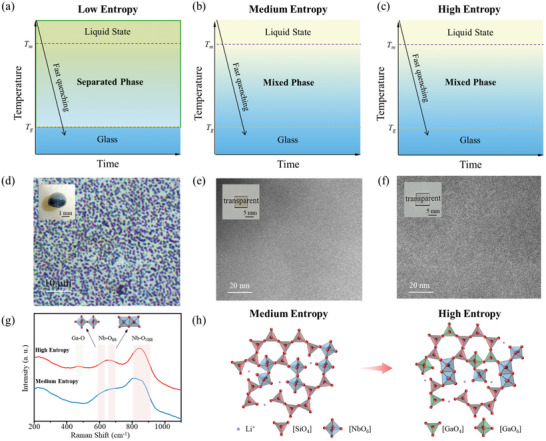
Entropy engineering for manipulation of glass formation. a–c) 3T diagrams for low (a), medium (b) and high (c) entropy niobosilicate glass systems. d–f) Morphologies of low (d), medium (e) and high (f) entropy niobosilicate glass and the insets are the photographs of the samples. g) Raman spectra of the medium and high entropy glass. h) The scheme illustrating the microstructures of medium and high entropy glass systems.

Firstly, the feasibility of entropy engineering strategy for control of glass formation was tested. In a typical example, the niobosilicate systems with different ∆*S*_mix_ were selected as the experimental candidate. As known, the binary system does not exhibit extensive solubility even at liquidus temperature (>*T*_m_), indicating that the ∆*S*_mix_ is too low to activate the entropy stabilized effect at liquidus temperature. The 3T (time‐temperature‐transition) diagram of the low entropy binary Nb_2_O_5_‐SiO_2_ system is shown in **Figure** **2**a, due to the deactivation of the entropy stabilized effect, homogeneous glass with single phase structure is not able to be fabricated theoretically. Experimentally, the binary sample was fabricated by laser‐assisted aerodynamic levitation technique (see Figure S1, Supporting Information and Experimental Section for details), which exhibits ultra‐fast cooling rate for hundreds of °C s^−1^. The melt was heated to >2000 °C by laser and followed quenched to room temperature. As shown in inset of Figure 2d, two prominent phases in macroscale can be observed, dark‐blue Si‐rich phase and buff‐yellow Nb‐rich phase. The microstructure of the dark‐blue phase is shown in Figure 2d, showing the inclusions of Nb‐rich phase texture in micrometer scale. These results are consistent with the report of Bright et al, who showed the immiscibility of the binary system.^[^ ^37^ ^]^ Confirmed by the XRD (as shown in Figure S2, Supporting Information), the low entropy system exhibits quite low glass formation ability with the uncontrollable crystallization of Nb_2_O_5_ (PDF 96‐152‐8724, 96‐210‐7338, 96‐210‐6535) even under such fast cooling rate.

In comparison, the triple medium entropy and quaternary high entropy niobosilicate systems with higher ∆*S*_mix_ are designed. The 3T diagrams of the medium and high entropy systems are shown in Figure 2b,c respectively. Due to the increasing of ∆*S*_mix_, entropy stabilized effect is activated at liquidus temperature, and transparent glass with homogeneous single phase can be potentially obtained by melt‐quenching process. As shown in the inset of Figure 2e,f, as expected, the melt‐quenching fabricated medium and high entropy niobosilicate glasses exhibit great transparency. The high‐resolution transmission electronic microscopy (HRTEM) and high‐angle annular dark field scanning transmission electronic microscopy (HAADF‐STEM) of the medium and high entropy niobosilicate glasses are shown in Figure 2e,f and Figure S3a,b (Supporting Information), respectively. Both systems exhibit similar homogeneous structure and no inclusions of secondary phase can be observed in nanoscale, indicating the preservation of entropy stabilized effect.

Since both the medium and high entropy systems can form homogenous glasses, their glass formation and anti‐crystallization ability are evaluated by differential scanning calorimetry, DSC (see Figure S4, Supporting Information for details). The glass transition temperature *T*_g_, the onset crystallization temperature *T*_c_ and Δ*T* (*T*_c_‐*T*_g_) are listed in **Table** **2**. It is found that the medium entropy exhibit higher *T*_g_, *T*_c_ and Δ*T* compared to that of the high entropy ones. Such results indicate that the medium exhibit highest glass formation and anti‐crystallization among the three systems.

**Table 2:** Characteristic temperature for medium and high entropy glasses.

	*T*_g_/^o^C	*T*_c_/^o^C	Δ*T*/^o^C
medium entropy	554	620	66
high entropy	530	565	35

To evaluate the micro‐configurations of the homogeneous medium entropy and high entropy glasses, the Raman spectrum of the high entropy glass was compared to the medium entropy one (Figure 2g). Vibrational bands of Ga‐O could only be observed in the range of 400–500 cm^−1^ from the high entropy systems,^[^ ^38^ ^]^ indicating the formation of [GaO_4_] and [GaO_6_] in high entropy system. The vibrational bands of the distorted [NbO_6_] octahedral covering 550–1000 cm^−1^ can be clearly observed from both the medium and high entropy systems. It can be found that both the vertex‐ (≈600 cm^−1^) and edge‐sharing (≈680 cm^−1^) [NbO_6_] are prevalent in the high entropy system, while the vertex‐sharing dominates in the medium entropy system.^[^ ^39^, ^40^ ^]^ Besides, the Raman band of terminal Nb─O bond at 800–900 cm^−1^ in high entropy system is greater than that in the medium entropy ones.^[^ ^39^, ^40^ ^]^ In this case, we suggest that the high entropy glass not only exhibit diversity of the types of element (Ga) and polyhedron ([GaO_x_]) but the diversity of the linkage of polyhedron (linkage between the [NbO_6_]) compared with the medium entropy one, indicating its intrinsic high mixed entropy property (Figure 2h).

The set of experimental outcomes show that the influence of mixed entropy on the glass formation ability for non‐metallic glass system would be twofold. On one hand, in the viewpoint of thermodynamics, higher ∆*S*_mix_ would lead to ealilier activation and preservation of the entropy stablized homogenous structure, which is benefit for glass formation. On the other hand, in the viewpoint of kinetic, the higher ∆*S*_mix_ would potential reduce the viscosity of the melt, which would lead to higher tendency of crystallization and might reduce the glass fromation ability.^[^ ^32^ ^]^ In our proposed system, components less than five can also activate the entropy stabilized effect. Once the entropy stabilized effect is activated, under the traditional quenching process, higher ∆*S*_mix_ would not lead to notable difference in the homogeneity of the non‐metallic glass.

### 2.3 Entropy Engineering for Manipulation of Phase Separation

Secondly, the possibility of entropy engineering strategy for control of phase separation was investigated. Reversibility is one of the features of the entropy‐driven transitions. Consequently, it is supposed that the homogeneous medium entropy and high entropy niobosilicate glasses would transform back to multi‐phase structure in the temperature between glass transition temperature (*T*_g_) and crystallization temperature (*T*_c_), if the entropy stabilized effect is failed to maintain at the temperature.

According to the DSC measurement (see Table 2 and Figure S4, Supporting Information for details), the glass transition temperature *T*_g_ was measured to be 554 and 530 °C for the medium entropy and high entropy niobosilicate glasses, respectively. The onset crystallization temperature *T*_c_ was measured to be 620 and 565 °C for the medium entropy and high entropy ones, respectively.

**Figure 3:**
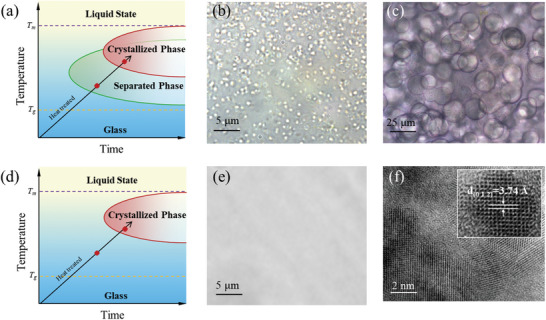
Entropy engineering for manipulation of phase separation. a) 3T diagram of medium entropy glass re‐heated to the temperature below *T_g_*. b) Optical microscopy image of medium entropy glass heat‐treated at 610 °C for 2h. c) Optical microscopy image of medium glass re‐heated to 650 °C for 2h. d) 3T diagram of medium glass re‐heated to the temperature below *T_g_*. e) Optical microscopy image of high entropy glass heat‐treated at 555 °C for 2h. f) TEM image of high entropy glass heat‐treated at 650 °C for 2h. The inset shows one typical grain.

As shown in **Figure** **3**a,d, the homogeneous medium entropy and high entropy niobosilicate glasses were heat‐treated at 610 and 555 °C for 2 h, respectively, which are 10 °C lower than their *T*_c_. The morphologies of the glasses after re‐heated are shown in Figure 3b,e. It can be found that the inclusions of droplet texture in micrometer scale can be clearly observed from the medium entropy sample after re‐heating, which is similar to that of the low entropy glass. On the contrary, homogeneity in the micrometer scale is maintained for the high entropic one, which is still amorphous (see Figure S5, Supporting Information for details).

The difference in structure‐temperature evolution behavior between the systems can also be readily explained by the entropy stabilized effect. From the viewpoint of thermodynamics, the entropy stabilized effect cannot be preserved at the temperature above *T*_g_ but lower than *T_m_*. But the higher ∆*S*_mix_ would reduce the tendency of phase separation. On the other hand, from the viewpoint of kinetic, long distance structures re‐arrangement would be activated at the temperature above *T_g_*, leading to the transformation from single‐phase to multiphase. Thus, in the high entropy system, entropy stabilized effect can still be better preserved at the temperature between *T*_g_ and *T_c_* due to its relative higher ∆*S*_mix_ and thus the homogeneous structure can still be maintained.

### 2.4 Entropy Engineering for Manipulation of Crystallization

Thirdly, the structure and phase evolution at the temperature above *T*_c_ were further studied. Both glasses were further heated at 650 °C for 2 h, which is above their *T_c_* to obtain the composite (red region in Figure 3a,d). The morphology of the medium entropy crystals‐in‐glass composite is shown in Figure 3c, showing the crystallized spheres with the size ≈20 µm. In stark contrast, the grains are estimated to be less than 10 nm for the high entropy system confirmed by the HRTEM (Figure 3f).

**Figure 4:**
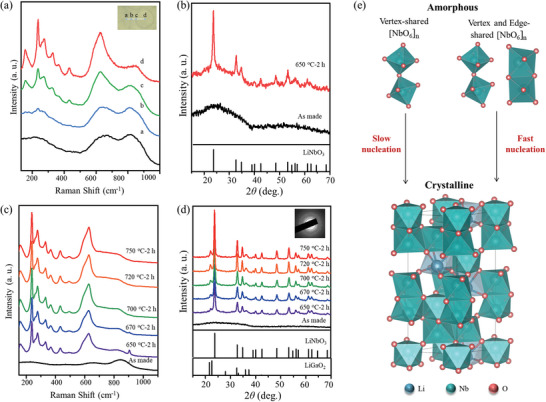
Entropy engineering for crystallization manipulation. a) Space selective Raman spectra of medium entropy crystals‐in‐glass composite heat‐treated at 650 °C. The inset shows the corresponding measured position. b) XRD patterns of medium entropy crystals‐in‐glass composite heat‐treated at 650 °C. c) Raman spectra of high entropy crystals‐in‐glass composite heat‐treated at the temperatures from 650 to 750 °C. d) XRD of high entropy crystals‐in‐glass composites heat‐treated at the temperatures from 650 to 750 °C. e) Schematically illustration of the crystallization process from vertex‐ and edged‐sharing [NbO_6_]_n_.

The crystallinity and phase of the precipitated crystal in crystals‐in‐glass composite were collaboratively studied via Raman spectra and X‐ray diffraction (XRD). The space selective Raman spectrum of the medium entropy crystals‐in‐glass composite fabricated via‐heat‐treatment at 650 °C is shown in **Figure** **4**a. Notably, the Raman spectra from position a to position d in the inset of Figure 4a are quite different from each other. Several sharp Raman peaks at 250, 441, and 630 cm^−1^ in position c and position d can be observed, which can be ascribed to the typical A_1_ TO and E TO vibrational modes of [NbO_6_] octahedron within the crystalline LiNbO_3_.^[^ ^41^, ^42^ ^]^ Differently, in position a and b, no obvious sharp peak can be observed in the Raman spectra, typical for amorphous niobosilicate glass. The results indicate the inhomogeneous distribution of the LiNbO_3_ microcrystals in the medium entropy crystals‐in‐glass composite. The XRD patterns of medium entropy crystals‐in‐glass composite shown in Figure 4b exhibit several sharp peaks, which can be indexed to the trigonal LiNbO_3_ (PDF 01‐074‐2240). It should be noticed that the broad peak resulting from the residual amorphous glassy phase is still obvious, indicating the low crystallinity in medium entropy crystals‐in‐glass composite. On the contrary, in high entropy crystals‐in‐glass composite, the sharp characteristic Raman peaks of LiNbO_3_ and their homogenous distribution in micro‐Raman mapping can be observed (as shown in Figure 4c; Figure S6, Supporting Information). The results confirm the homogeneous dispersion of the LiNbO_3_ nanocrystals in the glass phase. The XRD of the high entropy crystals‐in‐glass composite is shown in Figure 4d. The sharp characteristic diffraction peaks and the absence of the broad peak in high entropy crystals‐in‐glass composite indicate quite higher crystallinity compared with the medium entropy one.

The significant difference of the crystallization behavior between the medium and high entropy glass systems, featured by the sharp contrast in crystallite size and crystallization nucleation rate, might be originated from their entropic properties. First, the preservation of entropy stabilized effect results in the difference in crystallite size. As discussed above, the entropy stabilized effect would not be better preserved at the temperature below *T*_m_ in medium entropy system, leading to the phase separation into micrometer‐sized Nb‐rich and Si‐rich region. The LiNbO_3_ crystal would prefer to grow from the Nb‐rich region, leading to the fast growth rate and formation of the microscale crystals. On the contrary, entropy stabilized effect can be maintained for higher degree at the temperature below *T*_m_ in high entropy system and micrometer‐sized phase separation would not occur, resulting in the homogenous separation of [NbO_6_] and [SiO_4_]. The [SiO_4_] networks can help to confine the growth of the LiNbO_3_ crystals, favorable to achieve nanometer sized mono‐dispersed grains. Second, the indirect effect brought by higher ∆*S*_mix_ contributes to the difference in nucleation rate. On one hand, as mentioned above, higher ∆*S*_mix_ system would exhibit lower viscosity, resulting in higher tendency of crystallization. On the other hand, higher ∆*S*_mix_ would lead to diversity in micro‐configuration, which might contribute to the formation of more efficient nucleation sites. Herein, the high entropy systems exhibit multiplex micro‐configurations compared with the medium ones, especially the appearance of edge‐sharing [NbO_6_]. As well known, the crystalline LiNbO_3_ is generally non‐stoichiometric due to its higher thermodynamically stability than the ideal stoichiometric one.^[^ ^43^, ^44^ ^]^ As shown in Figure 4e and Figure S7 (Supporting Information), the non‐stoichiometric crystalline LiNbO_3_ is composed with both vertex‐ and edge‐sharing [NbO_6_]. The [NbO_6_] with multiplex linkage states might act as more efficient nucleation site compared to the single vertex‐sharing one, due to the similarity in configuration to the non‐stoichiometric crystalline LiNbO_3_. That is to say, high entropy system would benefit faster local re‐arrangement of the structure from amorphous to crystalline kinetically, but elements aggregation would be reduced and the growth of the grain would be confined thermodynamically, leading to its unique faster and nanocrystallization behavior.

Benefiting from the unique nanocrystallization habit brought by its high entropy property, the crystallinity of the high entropy crystals‐in‐glass composite can be further enhanced via higher temperature heat‐treatment. The Raman and XRD of high entropy crystals‐in‐glass composite heat‐treated at 650 to 750 °C for 2 h are shown in Figure 4c,d. With the increase of the heat‐treatment temperature from 650 to 720 °C, the characteristic peaks for crystalline LiNbO_3_ in XRD and Raman spectra become more intense and the peaks of residual glassy phase are almost completely suppressed, indicating the improvement of crystallinity. The characteristic peaks for crystalline LiNbO_3_ in XRD and Raman spectra of high entropy crystals‐in‐glass composite heat‐treated at 750 °C are similar to that in the sample heat‐treated at 720 °C, indicating the confined crystallization of LiNbO_3_ nanocrystals has been achieved. Moreover, all of the high entropy crystals‐in‐glass composites exhibit great transparency (see Figure S8, Supporting Information for details), which might bring new opportunities for photonic applications.

### 2.5 Nonlinear Optical Response in High Entropy LiNbO_3_ NiG Composite

LiNbO_3_ is one of the most excellent optical nonlinear materials with the maximum second order nonlinear coefficient of ≈30 pm V^−1^. The high entropy LiNbO_3_ nanocrystal‐in‐glass (NiG) composite embedded with dense LiNbO_3_ nanocrystals loading is supposed to exhibit efficient second order nonlinear response. To estimate the effective nonlinear coefficient of the high entropy NiG composite, the generation of nonlinear second harmonic (SHG) from the high entropy NiG heat‐treated at 720 °C is measured. The commercial *β*‐BBO (BaB_2_O_4_) nonlinear crystal was employed as reference in the standard measurement.

The intensity generation of SHG can be expressed as

(3) $$I_{\mathrm{SHG}}=d_{\mathrm{eff}}\;{I_{\mathrm{FUND}}}^{2}$$

in logarithmic form, it can be described as

(4) $$\mathrm{lg}I_{\mathrm{SHG}}=2\mathrm{lg}I_{\mathrm{FUND}}+\mathrm{lg}d_{\mathrm{eff}}$$

where *I*_SHG_ and *I*_FUND_ are the intensity of SHG and fundamental laser source, respectively, *d*_eff_ is the effective second order nonlinear coefficient.

**Figure 5:**
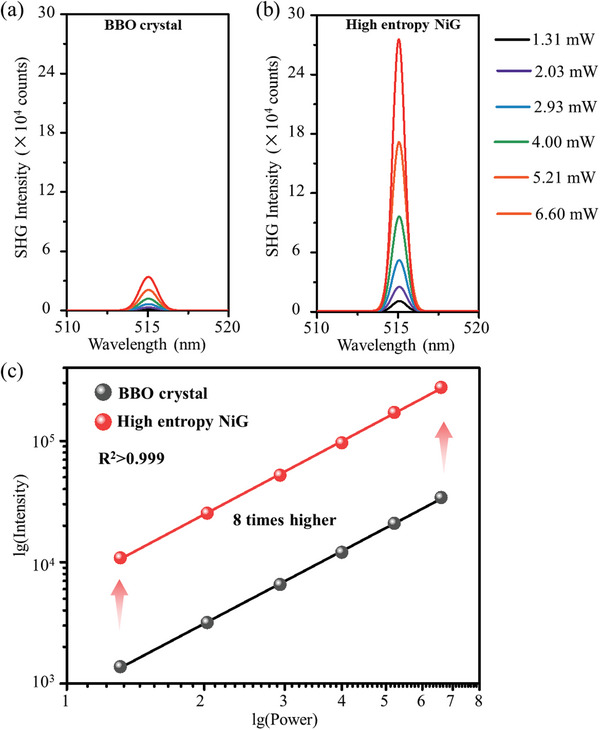
Nonlinear second harmonic response in high entropy NiG composite. a,b) Generated SH intensity from *β*‐BBO crystal (a) and high entropy NiG composite (b) with irradiation by the 1030 nm femtosecond laser. c) Input power dependent SH intensity of *β*‐BBO crystal and high entropy NiG composite.

As shown in **Figure** **5**a,b, obvious SHG emission at 515 nm can be detected from both *β*‐BBO crystal and the high entropy NiG composite under the irradiation by the 1030 nm femtosecond laser. The fundamental laser source intensity dependent SHG emissions are shown in Figure 5c. By linear fitting, the logarithmic intensity of SHG shows great linearity to the logarithmic power of the input fundamental light source with slop equals to 2 and R^2^>0.999. The fitting curves are in excellent agreement with the Equation (4). Thus, by comparing the intercept of the fitting curves, it can be estimated that the nonlinearity is ≈8 times higher than that of the commercial *β*‐BBO (*β*‐BaB_2_O_4_) nonlinear crystals.

### 2.6 Photonic Application for Ultra‐Short Pulse Monitoring by Using High Entropy LiNbO_3_ NiG Composite

**Figure 6:**
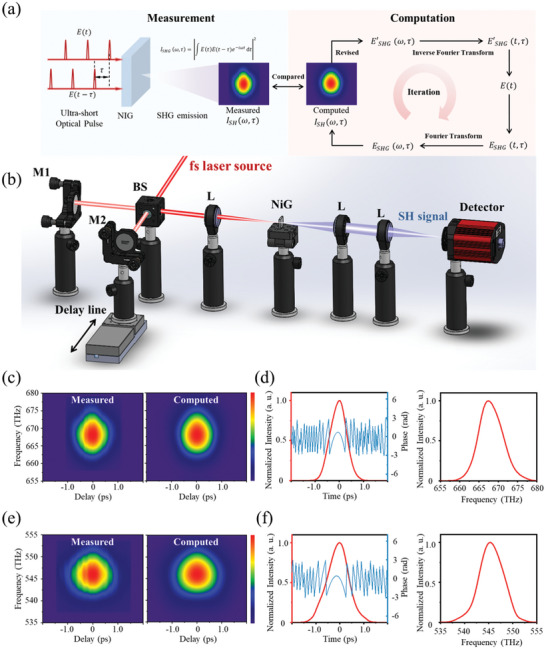
Photonic application for ultra‐short pulse monitoring. a) Principle of FROG measurement. b) Measurement setup for FROG trace by using high entropy NiG composite as nonlinear medium. c) Measured (left) and computed (right) FROG trace of the 900 nm femtosecond pulse. d) Temporal pulse intensity and phase (left) of the 900 nm femtosecond pulse and frequency dependent intensity of generated SH (right) excited by 900 nm femtosecond laser. e) Measured (left) and computed (right) FROG trace of the 1100 nm femtosecond pulse. f) Temporal pulse intensity and phase (left) of the 1100 nm femtosecond pulse and frequency dependent intensity of generated SH (right) excited by 1100 nm femtosecond laser.

The attractive nonlinear optical properties of the constructed high entropy high entropy LiNbO_3_ NiG composite prompt us to explore its practical photonic applications. As a typical example, its potential for ultra‐short optical pulse duration and phase measurement via FROG (Frequency‐resolved optical gating) method was explored. The principle for FROG measurement is schematically shown in **Figure** **6**a. Two ultra‐short pulses with certain delay are injected into the nonlinear media to generate nonlinear SHG. The frequency and delay dependent SHG intensity would be recorded as the FROG trace. Once we obtain the FROG trace, a standard iterative phase retrieval algorithm can be used to reconstruct the intensity and phase of the pulse. The algorithm starts with a guessed initial solution which satisfies nonlinear optical constraints. The computed FROG trace would be generated based on the solution and be compared to the measured one.^[^ ^45^ ^]^ The algorithm would repeat until it obtains the solution that satisfies both the optical constraints and measured data. As a result, the temporal pulse intensity, phase and the frequency dependent intensity would be constructed based on the final solution. The detailed setup for FROG trace measurement is shown in Figure 6b. The ultra‐short femtosecond pulse was split into two beams by the 50:50 beam splitter (BS). The delay between the two split pulses was set by a stepping‐motor and focused into the 1.5 mm thick high entropy NiG composite by the lens (L) to generate the nonlinear SHG. For each delay, the SH was detected by the spectrometer to generate the FROG trace.

Figure 6c,e shows the FROG traces excited by the femtosecond pulse with the wavelengths centered at 900 and 1100 nm respectively. It can be observed that the measured FROG traces excited by both pulses are intense. This indicates that the high entropy NiG composite enables to achieve efficient and broadband SHG. The final computed FROG trace shows an excellent match between the measured and the computed trace, confirming the accuracy of the rebuilt results. Retrieved from the measured FROG traces, the temporal pulse intensity and phase of the 900 and 1100 nm ultra‐short pulses as well as the frequency dependent intensity of generated SHG excited by 900 and 1100 nm pulses can be rebuilt and displayed in Figure 6d,f, respectively. It should be noted that FROG measurement is generally performed by the nonlinear crystal such as *β*‐BBO as nonlinear media. To compensate for phase mismatch, the irradiated angle of the pulses against the nonlinear crystals needs to be adjusted carefully to generate efficient SHG, which is strictly dependent on the wavelength of the input pulse. Due to the unique random domain structure of NiG composites, the efficiency of SHG is irradiated angle and wavelength insensitive.^[^ ^46^, ^47^ ^]^ Furthermore, the NiG composites also exhibit higher humidity and temperature stability compared to that of nonlinear crystals. Thus, the high entropy NiG composite shows competitiveness to the commercial nonlinear crystal, such as *β*‐BBO in the application of FROG measurement.

## 3 Conclusion

In summary, we have expanded the concept of entropy engineering from alloys and ceramics to non‐metallic inorganic glass. The comparison of the concept between alloys, ceramics and non‐metallic glass has been discussed theoretically. As a typical example, the activation and preservation of entropy stabilized effect of the niobosilicate glass system at different temperature have been studied (as shown in **Table** **3**). Furthermore, the micro‐configurations of the glass with different entropic property have also been analyzed. Importantly, the NiG composite with high nonlinearity has been developed based on the entropy engineering strategy, with nonlinearity 8 times higher compared with the commercial nonlinear crystals *β*‐BBO. Furthermore, we have demonstrated the applications of the constructed high entropy NiG composite for precise measurement of the duration and phase of ultra‐short femtosecond pulse. Our findings gained from our experimental and theoretical results are supposed to provide new directions for both materials science and photonic applications.

**Table 3:** Summary of the activation and preservation of entropy stabilized effect for niobosilicate glass system at different temperatures in quenching‐heat treating process.

temperature	low entropy	medium entropy	high entropy
*T*>*T_m_*	deactivation	activation	activation
*T*<*T_g_*	deactivation	preservation	preservation
*T_c_* >*T*>*T_g_*	deactivation	de‐preservation	preservation

## 4 Experimental Section

### Materials Synthesis

To design the non‐metallic glasses with significant differences in entropy, the independent components are supposed to exhibit different properties with each other. Herein, four kinds of components are selected based on the field strength factor (*F*): classical modifier (*F*<0.4 Å^−2^, e.g., Li_2_O), classical former (*F*>1.3 Å^−2^, e.g., SiO_2_), classical intermediate (0.4<*F*<1.0 Å^−2^, e.g., Ga_2_O_3_) and high polarized component (1.0<*F*<1.3 Å^−2^, e.g., Nb_2_O_5_).

Precursor glass was synthesized by the classic melt‐quenching and laser‐assisted aerodynamic levitation technique. Stoichiometric amounts of high‐purity raw materials: Li_2_CO_3_ (Aladdin 99.998%), Nb_2_O_5_ (Cdalfa 99.99%), Ga_2_O_3_(Aladdin 99.99%), and SiO_2_ (Macklin 99.99%) were weighed to give 40 g powder batches. The compositions of the low entropy samples were tuned to be (mol%)24Nb_2_O_5_‐76SiO_2_. The powder batches were homogeneously mixed and mashed into small blocks with the size ≈2×2×2 mm^3^. A small block was put on the sample stage of the aerodynamic levitator furnace, heated by CO_2_ laser, and N_2_ was introduced to make it suspended and fired homogeneously. The laser power was controlled in the range of 300–500 kW and the sample would be heated for >2000 °C. The sample would be quenched by turning the laser off and be cooled rapidly to room temperature at hundreds of °C s^−1^. After cooling, a sphere sample with the can be prepared the compositions of the medium and high entropy samples were tuned to be (mol%)34Li_2_O‐24Nb_2_O_5_‐42SiO_2_ and 34Li_2_O‐24Nb_2_O_5_‐24SiO_2_‐18Ga_2_O_3_, respectively. The powder batches were homogeneously mixed and melted in a Pt‐Rh crucible at 1500 °C for 45 min. The melts were poured on a stainless steel plate at room temperature and pressed by another plate to obtain samples with a thickness of ≈1.5 mm. The crystals‐in‐glass composites were elaborated through in situ crystallization from precursor glass by heat‐treatment. Precursor glass was obtained by heat‐treatment at the specific temperature (>*T*_c_) for 2 h.

### Material Characterization

*T_g_* and *T_c_* of the precursor glasses were measured using DSC (NETZSCH STA 449C) by heating at 10 °C min^−1^ in air. XRD measurements were performed using a diffractometer (X'Pert Powder, PANalytical, Netherlands) equipped with Cu Kα1 as the incident radiation source. Raman spectra were recorded by a Raman Renishaw InVia spectrometer with a 532 nm laser source. Microstructures were analyzed by optical microscopy and TEM. The microscale microstructure was analyzed by an optical microscope (Nikon, Eclipse, Japan) equipped with a charge coupled device (CCD, Nikon, DS‐Fi1, Japan). The nanoscale microstructure was analyzed by HRTEM (Tecnai G2 F20s‐twin, FEI, USA) and HAADF‐STEM (Themis Z, FEI, USA).

### Nonlinear Light‐Matter Interaction Characterizations

To measure the SHG efficiency, a femtosecond laser centered at 1030 nm was employed as a fundamental pump. The pump laser is focused by a 50× objective lens on the NiG composite and the *β*‐BBO samples. Under the excitation, the scattered and reflected SHG signal from the samples would be collected by the same objective lens and be detected by the spectrometer.

## Conflict of Interest

The authors declare no conflict of interest.

## Author Contributions

S.Z. conceived the idea and initiated the research. S.Z. and X.J. designed the experiments. X.F., G.G. and J.T. performed the experiments and collected the data. X.F., Q.L., Y.Y., J.T., Z.L., and J. Q. analyzed the data and discussed the results. X.F. wrote the manuscript, and S.Z., X.J., and J.Q. revised and commented on it.

## Supporting information



Supporting Information

## Data Availability

The data that support the findings of this study are available from the corresponding author upon reasonable request.
