# Supplementary material for: Entropy‐Mediated Crystallization Manipulation in Glass
Source: Adv Sci (Weinh). 2024 Dec 16;12(6):2411861. doi: 10.1002/advs.202411861 (PMC11809423; doi:10.1002/advs.202411861)
Supplement: Supplementary file 1 — Supporting Information [file ADVS-12-2411861-s001.docx]

Supporting Information

**Entropy-mediated crystallization manipulation in glass**

*Xu Feng, Guanfeng Gao, Quanhua Lin, Yongkang Yang Jiajia Tan, Ziang Liu, Jianrong Qiu, Xiaofang Jiang & Shifeng Zhou**


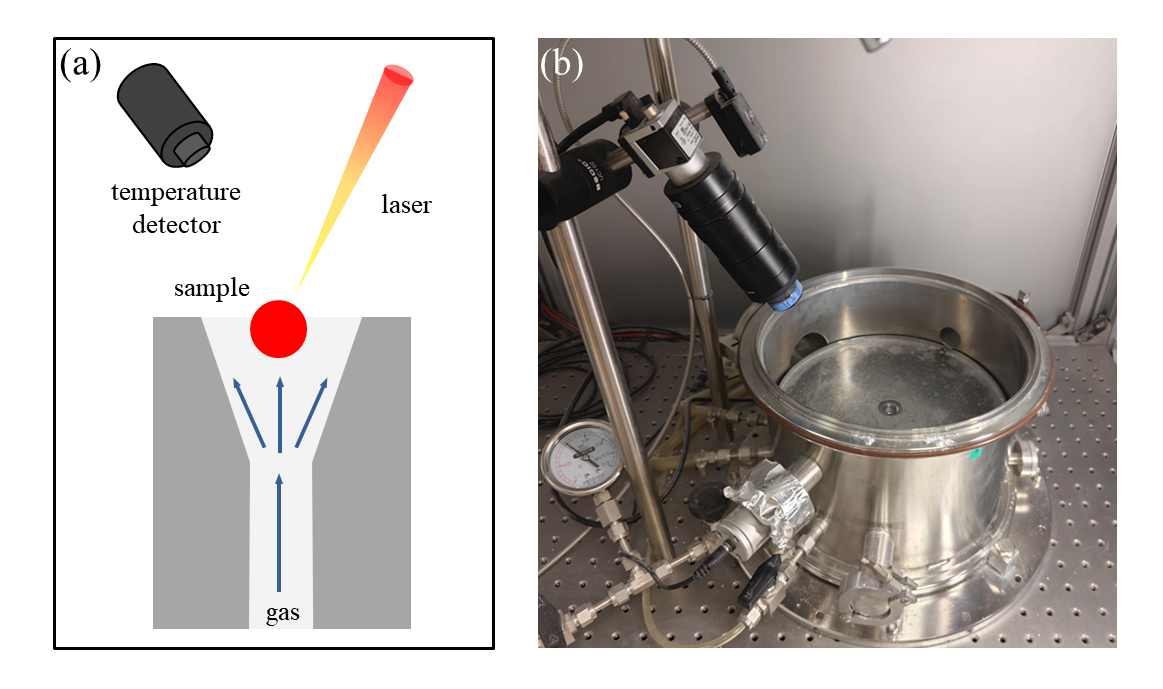


Figure S1. (a) The scheme of laser-assisted aerodynamic levitation technique. (b) The setup of the aerodynamic levitator furnace.


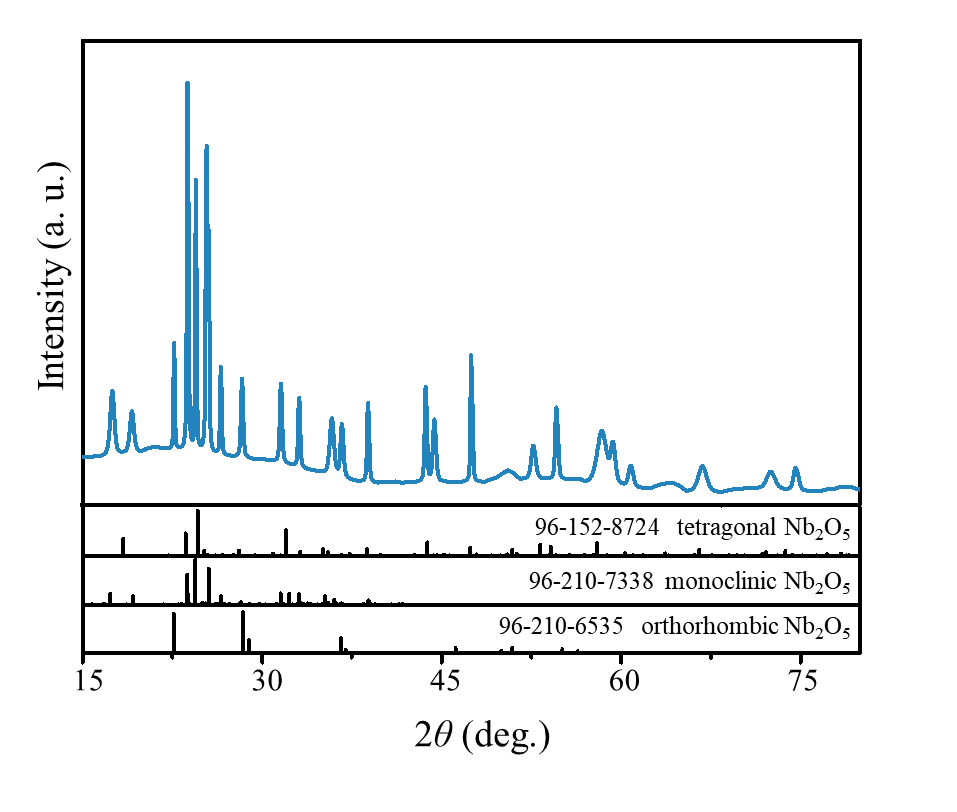


Figure S2. XRD of the as made low entropy sample


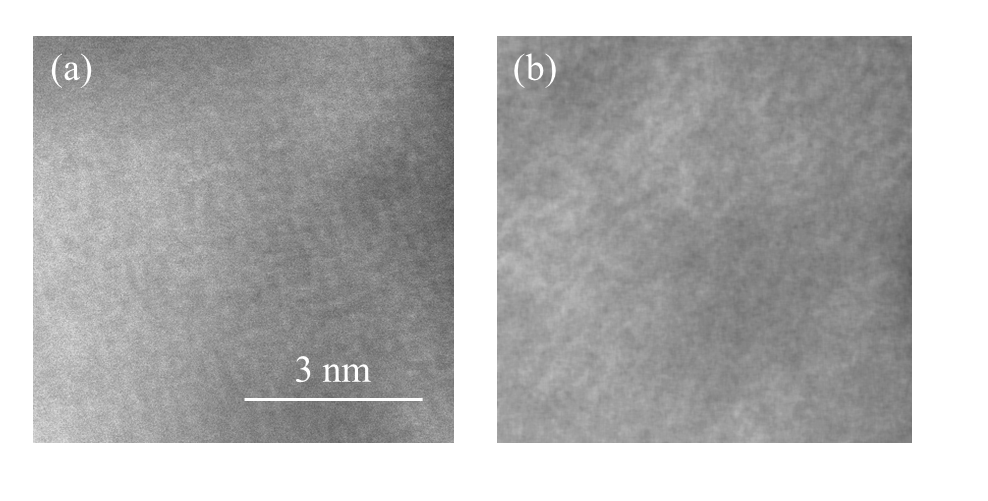


Figure S3. HADDF-STEM of a) medium entropy and b) high entropy glasses


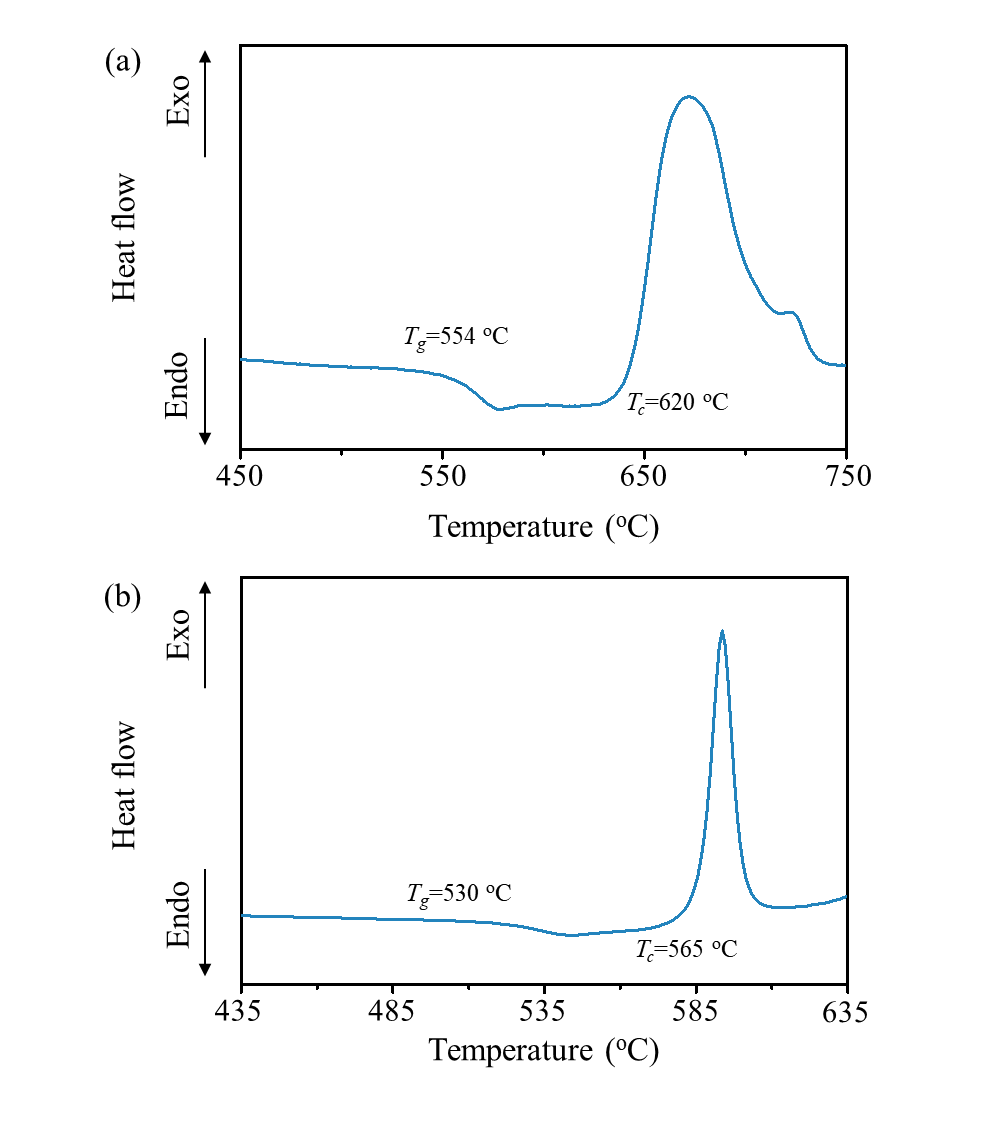


Figure S4. DSC curves of the a) medium entropy and b) high entropy glass.


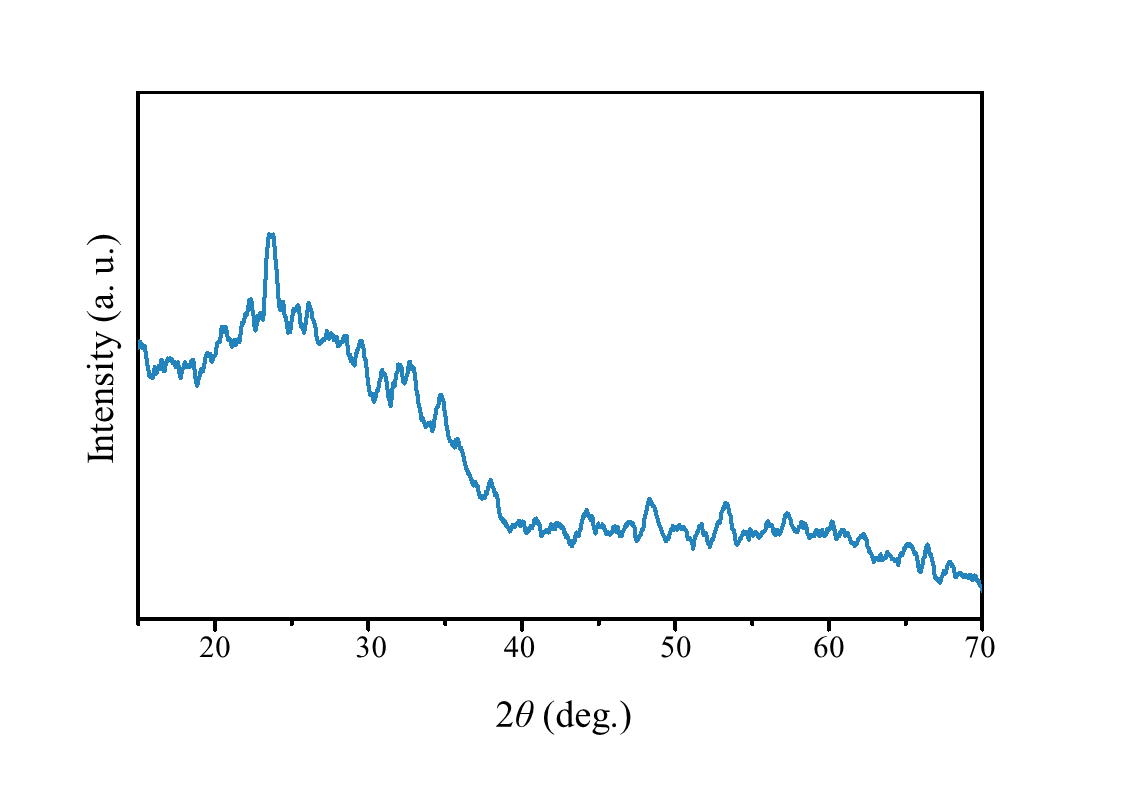


Figure S5. XRD of the high entropy glass heat treated at 555 ^o^C for 2 h.

**
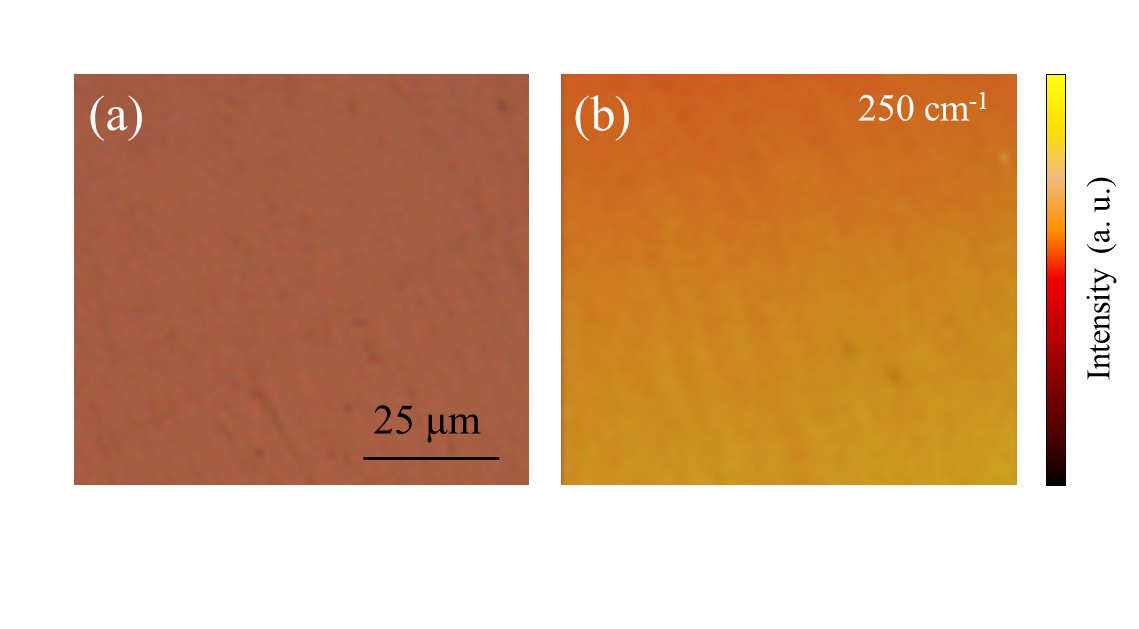
**

Figure S6. Micro-Raman mapping of the high entropy NiG composite. a) Micro-optical microscopy of the mapping region. b) Micro-Raman mapping of the characteristic band of crystalline LiNbO_3_ at 250 cm^-1^.

**
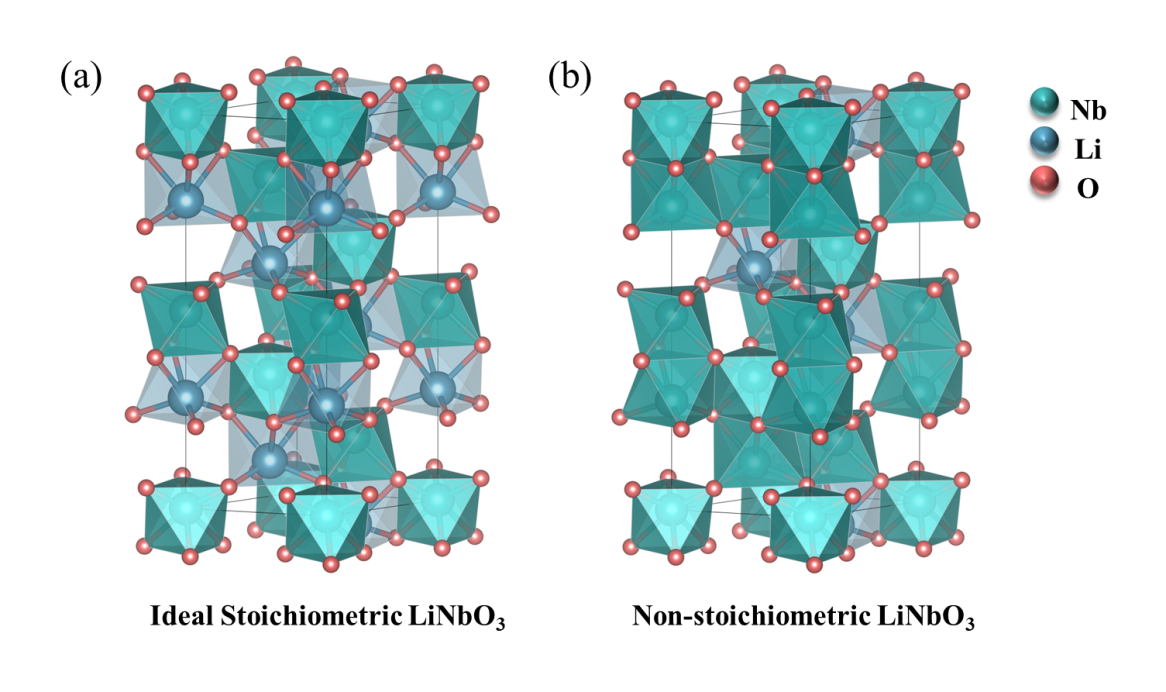
**

Figure S7. Structure of LiNbO_3_ crystals. a) The lattice unit of ideal stoichiometric LiNbO_3_, which is composed with vertex-sharing [NbO_6_] and edge-sharing [NbO_6_]-[LiO_6_] pair. b) The lattice unit of non-stoichiometric LiNbO_3_ with Nb_Li_^4+^ defects, which is composed with both vertex-sharing and edge-sharing [NbO_6_].


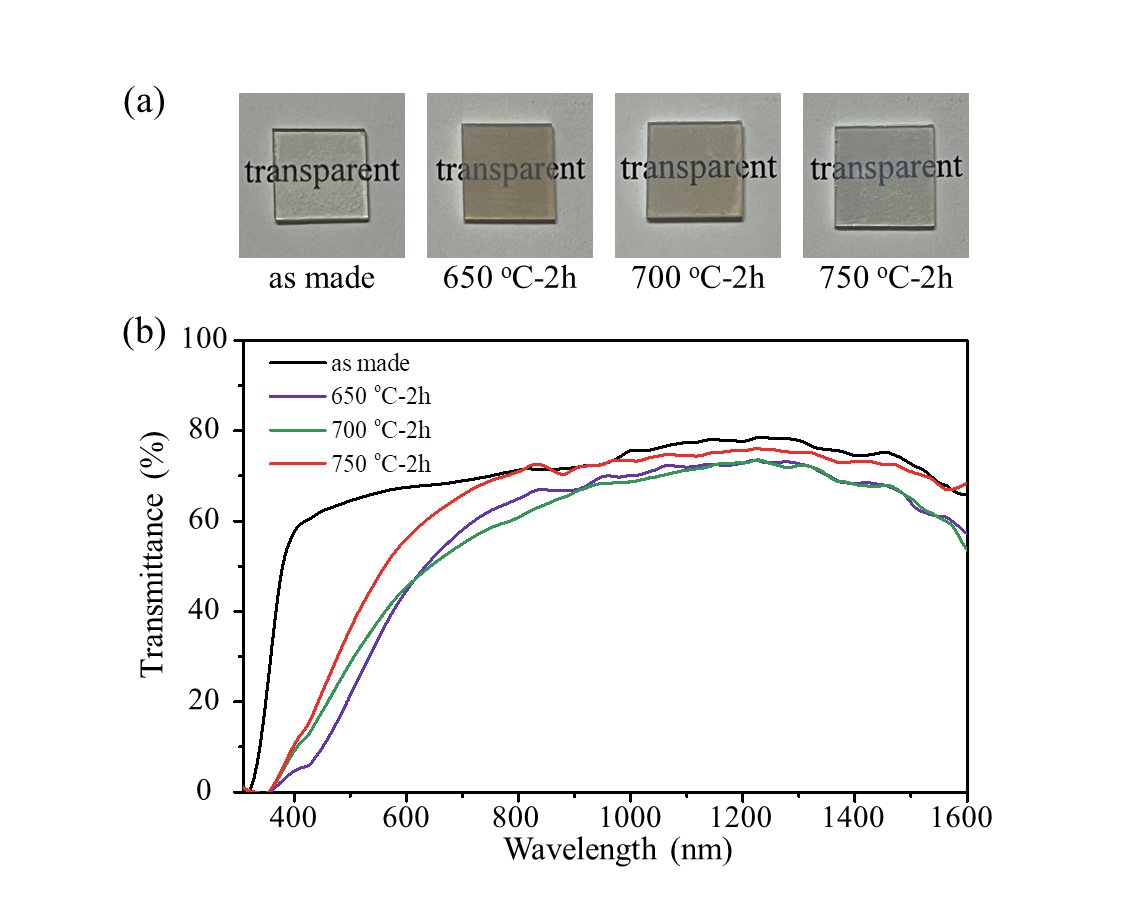


Figure S8. Transparency of the high entropy nanocrystals-in-glass composite. a) Photographs of the high entropy NiG composites fabricated at different temperatures. b) Transmittance spectra of the high entropy NiG composites.
